# Unventilated Indoor Coal-Fired Stoves in Guizhou Province, China: Reduction of Arsenic Exposure through Behavior Changes Resulting from Mitigation and Health Education in Populations with Arsenicosis

**DOI:** 10.1289/ehp.9273

**Published:** 2007-01-09

**Authors:** Dong An, Dasheng Li, Yin Liang, Zhengjin Jing

**Affiliations:** Guizhou Center for Disease Control and Prevention, Guiyang, Guizhou Province, People’s Republic of China

**Keywords:** arsenic, arsenicosis, coal, China, Guizhou, health education, mitigation

## Abstract

We the report results of a coordinated mitigation effort aimed at reducing arsenic (As) exposure in three counties of Guizhou province, China. Mitigation occurred in 2005 and encompassed 21 villages with 47,000 inhabitants, who were exposed to high levels of As in their diet through consumption of As-contaminated chili peppers and corn dried over unventilated stoves that burned coal containing high levels of As. The coal was mined by villagers from local pits. Inhalation of air that contained high levels of As contributed to approximately 25% of the daily As intake of 6–9 mg. Before mitigation, a baseline survey of 45,364 residents in 2004 identified more than 2,800 individuals with arsenicosis. The survey also found that many residents were aware of the health effects of As in general but lacked in-depth understanding of the link between coal use and arsenicosis. Consequently, an overwhelming majority (> 95%) continued to use high-As coal. This survey provided the basis for a health education campaign that promoted lifestyle changes coupled with the shutting down of local coal pits and the installation of 10,000 new stoves with chimneys for ventilation. The cost of the mitigation was about 4 million Yuan RMB (US$500,000) and was financed mostly by the government. A postmitigation response survey in 2005 found that > 85% of the residents now associate the use of coal with arsenicosis; > 90% correctly learned to operate the new ventilated stoves; and > 90% dry corn and chili peppers outdoors in the sun. Urinary As concentrations in the region decreased from 0.198 ± 0.300 mg/L (*n* = 144) in 2004 to 0.049 ± 0.009 mg/L (*n* = 50) in 2005 in individuals with arsenicosis (*p* < 0.01), which is consistent with the behavior changes.

Food contamination and air pollution caused by domestic coal burning has been linked to severe arsenicosis endemics in Guizhou Province, China (Finkelman et al. 1999; Zheng et al. 1999). It was estimated that approximately 200,000 residents in Xingren, Xingyi, and Anlong counties of Qianxinan prefecture were at risk of exposure to high levels of As (Liu et al. 2002). Recent surveys of the three counties studied by the Guizhou Centers for Disease Control (CDC) found that approximately 47,000 people were exposed to high concentrations of As. In this region, more than 2,800 individuals with arsenicosis were identified (An and Li 2005; Li and An 2005). Typical clinical symptoms included brown pigmentation and depigmentation, maculae on the skin in the unexposed parts of the body, hyperkeratosis on palms of the hands and soles of the feet, cankers, and skin cancer (Li et al. 2000). Cancers of internal organs, such as lung and liver cancers, have been common (Li et al. 1999a, 2004). Congenital deformities due to As exposure and manifested in adolescents were also observed (Li et al. 2005b). The devastating health effects of arsenicosis became an enormous burden for individuals and households, affecting their ability to work. Consequently, the villagers continue to live in extreme poverty, even by Chinese rural standards (Finkelman et al. 2003; Liu et al. 2002).

As early as the 1960s, large but unknown numbers of individuals with arsenicosis were found in the Bazhishang village of Zhijin county and the Jiaole village of Xingren county. Induction of arsenicosis in these people was attributed to indoor the burning of high-As coal that was mined by villagers in small coal pits (An et al. 1996). Since the 1960s, the local government has implemented several mitigation measures, such as restrictions on the mining of high-As coal and treatment of the patients with arsenic-chelating drugs (Zhou et al. 1998). However, these mitigation measures were not effective, in part because of the chaotic political environmental in the era of the Cultural Revolution, circa 1966–1976. Compared with other areas in Guizhou, restrictions on mining high-As coal were most effective in Zhijin county because of the small geographic area and the limited number of coal pits (Li et al. 1999b).

By the early l990s, the arsenicosis endemic spread from Jiaole village of Xingren county to other villages in the same county, and to two more counties, Xingyi and Anlong (Li et al. 1999a; Zhang et al. 2003). The number of arsenicosis cases increased significantly in Jiaole village, as the exposure time by then was more than 20 years (An and Li 2005; Li and An 2005). Again, the government began shutting down the mines that produced high-As coal, providing treatment of symptoms for some of the individuals with arsenicosis, and installing stoves with chimneys in some of the households. Unfortunately, most residents were still unaware of the grave health consequences of domestic coal use. Therefore, they continued to mine and use the high-As coal as a source of free fuel. Furthermore, many were too poor to afford a fraction of the cost required to install a stove with a chimney or did not know how to use the stoves properly. Therefore, As contamination of corn and chili peppers dried over the unventilated stoves and of the indoor air continued and may have worsened (An and Li 2005; Li and An 2005). New cases of arsenicosis continued to appear in the late 1990s (Li et al. 1999a, 2000).

Recently, improved economic development in China and the increasing awareness of arsenicosis by international organizations such as UNICEF (United Nations International Children's Emergency Fund) allowed a more concerted mitigation effort to reduce As exposure in the As-endemic areas of Guizhou. In this article, we describe evidence that supports reduction of As exposure in the exposed population through implemention of mitigation measures that would eliminate the source of exposure, with a strong emphasis on health education that promotes awareness of the link between domestic coal use and arsenicosis.

## Methods

### Extent of mitigation

Mitigation took place in 21 villages in nine towns in the arsenicosis-endemic counties of Xingren, Xingyi, and Anlong in southwestern Guizhou. The endemic region comprising 21 villages had a population of 47,000. For the three counties surveyed, the population estimates were as follows: Xingren, 19,000 for 3 villages surveyed in three towns; Anlong, 18,000 for 12 villages in three towns; and Xingyi, 10,000 for 6 villages in three towns.

### Baseline survey

From December 2004 to April 2005, the Guizhou CDC undertook a survey that included a questionnaire of factors that influence lifestyle and behavior (building type, fuel source, coal-burning stove style, staple food, food dehydration and storage, and food washing before cooking), a questionnaire of awareness of the health effects of arsenicosis, and a medical examination to assess the status of arsenicosis in residents. The procedure to obtain informed consent before medical examination was reviewed and approved by the Institutional Review Committee of the Guizhou CDC. The survey teams comprised provincial and county CDC professionals and doctors from hospitals in each town. Training of the survey team members was conducted at the county CDC before the survey was taken. Ten households randomly selected in 3 villages that were also randomly selected in each town were surveyed for behavior factors. A total of 270 households were included in the survey and a total of 141 households were asked about their awareness of the health effects of arsenicosis. This sample represents about half the 10 households selected at random in 3 villages. In addition, an awareness questionnaire was completed by 1,393 primary school students from every class from Grades 3–6 in the town center primary school, with one primary school located in each town. The same survey team examined 45,364 residents from all villages for symptoms of arsenicosis following the Chinese National Standard of Arsenicosis Diagnosis (WT-2001; Sun et al. 2004). This is 96% of the residents examined, as the total population of the region is 47,000. A small number of samples of indoor air (*n* = 37), food dried over stoves (*n* = 70), drinking water (*n* = 17), and urine (*n* = 60) were collected by the same survey team.

### Arsenic analysis

We used the National Standard methods to determine the concentrations of As in the samples and followed standard protocols of the Guizhou CDC for environmental sample analysis in China. The methods used to analyze indoor air, food, drinking water, urine, and coal were GB 8912-1988, GB/T 5009.11-1996, GB/T 8538-1995, WS/T 28-1996, and GB/T 3058-1996, respectively (Ministry of Health 2007). Here, GB (GuoBiao) translates to “National Standard Method.” Silver dithiodicarbomate spectrometry (DDCAg) to quantify As after the samples have been treated. Briefly, the indoor air samples collected by filtration (GB 8912-1988) were collected on 3 consecutive days. The sampling for each day consisted of three replicate samples from each household, and the mean concentrations of the three replicate samples over 3 days were reported. The food samples, including corn and chili peppers, were hung over the coal fire in the kitchen for about 3 months before collection. The food samples were dried in an oven without washing, ground to powder, and digested with acids (GB/T 5009.11-1996). The drinking water samples were collected from the wells—the only water source for the villages—and were acidified (GB/T 8538-1995). Samples of coal were obtained from the residents and from selected coal pits that were easily accessible from the villages. The coal samples were also dried in an oven, ground to powder, and digested in acid (GB/T 3058-1996). The urine samples were collected from individuals who had submitted to medical examinations and stored at 4°C in the field and −20°C on returning to the CDC for analysis (WS/T 28-1996). Urine and water samples were usually analyzed within 1 month of sample collection.

### Mitigation measures

#### Health education

Health education in the endemic regions was aimed at primary school students in grades 3–6, middle school students in grades 7–12, and heads of households. Based on the results of the baseline survey questionnaires of behavior factors and awareness, we were able to identify the missing gaps in the core knowledge about the health effects of As resulting from the domestic use of coal. Educational materials such as curriculum material for classroom teaching for students, brochures, video compact disc (VCD) programs, posters, bulletin boards, signs, and slogans were developed and distributed in schools and by village health clinics. In each village, a community-based health education campaign was conducted. The campaign included village group meetings; weekly market-day health consultations; distribution of health education brochures to villagers visiting health clinics and hospitals; door-to-door visits and consultations by the CDC team members; cable television broadcast of VCD programs; and the posting of slogans and the updating of village bulletin boards. Curriculum materials explaining the link between the contamination of food and air by unventilated indoor coal-fired stoves and arsenicosis were incorporated into classroom teaching in local primary and middle schools. Students participated in designing the bulletin boards posted in their villages, wrote essays on topics of arsenicosis, and participated with CDC professionals in door-to-door visits to their neighbors. Schools also hosted many parent conferences to reinforce the educational campaign in the villages. In summary, the approach of health education combines community outreach with abundant individual consultation. By promoting health education to students and heads of households, we expanded the effect to a much greater reach in the community.

#### Shutting down the mines producing high-As coal

Local governments in counties, towns, and villages took charge of shutting down coal pits that produced high-As coal in the endemic arsenicosis areas. Explosives were used to render the coal pits unusable before the pits were sealed. Permanent warning signs made of concrete blocks or stones were posted in the pits where villagers mined coal known to contain high levels of As. We recognize that this does not exclude new pits producing high-As coal in the future but regulations on such mines do not currently exist.

#### Installation of ventilated stoves

New stoves with chimneys, which are designed to meet most of the cooking and heating needs of residents, were installed in all households in the areas with endemic arsenicosis. The central government provided major subsidies for the installation, with a minor contribution by each household. The government provided a total of 3,700,000 Yuan RMB (~ US$500,000), and the residents paid a total 216,000 Yuan RMB (~ US$28,000). The total financing was 3,916,000 Yuan RMB (~ US$506,000) and was used mostly for the purchase and installation of 10,000 new stoves, and for payment of health education costs. In addition, the use of commercial coal briquettes containing low levels of As to further reduce As exposure was also encouraged.

### Collaboration among government agencies

An executive office, was formed to provide leadership on As mitigation at the province, prefecture, county, town, and village levels. This office coordinated and promoted the collaborations of the Departments of Finance, Health, Education, Agriculture, Communication and Outreach, and other related departments. The office consulted these departments for the implementation of the As mitigation program that set specific targets for each government department. The specific aims developed by the executive office were incorporated into the annual management plan of these departments at the province, prefecture, county, town, and village levels. The head of each of these departments signed an agreement to fulfill the mitigation targets. In addition, the departments provided technical and management expertise, quality control, and assessment and evaluation.

## Results

### Prevalence of arsenicosis

The results of our baseline survey conducted in 2004 identified two new villages with arsenicosis, or one more endemic town in Anlong, not previously identified by the survey in 1994 (Table 1). In Xingren, four new villages with arsenicosis, or two more towns, were found in the 2004 survey (Table 1). In Xingyi, the number of endemic villages and towns remained the same (Table 1). Most of the arsenicosis patients were from Xingren county, with the number of cases increasing from 1,565 in 1994 to 2,250 in 2004, which corresponds to a total population of 14,685 in 1994 and 20,747 in 2004 (Table 1). Thus, the prevalence rate of arsenicosis in Xingren was comparable at approximately 10% in both 1994 and 2004. The prevalence rate of arsenicosis in Anlong was approximately 3% in both 1994 and 2004. However, the prevalence rate in Xingyi was approximately 7% in 1994 and significantly lower at approximately 4% in 2004.

### Arsenic exposure

Arsenic concentrations in coal averaged hundreds of milligrams per kilogram in three counties (Table 2). Indoor open-stove burning of high-As coal results in high concentrations of As in indoor air—approximately 0.2 mg/m^3^ (Table 2). Corn dried over the open stoves using this coal had As concentrations averaging a few milligrams per kilogram (Table 2). Similarly, dried chili peppers contained enormous amounts of As, averaging hundreds of milligrams per kilogram (Table 2). The high levels of As exceeded the Chinese National Standard of Arsenic for Food (GB4810-1994; Ministry of Health 2007) by a factor of 100–10,000. In comparison, As concentrations in six coal samples from Xingren county, where no cases of arsenicosis were found, averaged approximately 11 mg/kg (Table 2). Not surprisingly, the As concentrations in corn, chili peppers, and indoor air are all orders of magnitude lower than those from the arsenicosis-endemic villages (Table 2). Concentrations of As in drinking water provided by community water supplies from groundwater wells are lower than that of the maximum permissible level of 50 μg/L (GB5749-1985; Ministry of Health 2007). Excluding drinking water, daily As exposure via food and air is estimated to be 6–9 mg (Li et al. 2002), with approximately 45% from chili peppers, 30% from corn, and 25% from air.

### Behavior factors and awareness

At the time of the baseline survey in 2004, an overwhelming majority of residents in the three arsenicosis-endemic counties were still using stoves without a chimneys to heat, cook, and dry corn and chili peppers (Table 3). Most of the corn and chili peppers were not washed before cooking (Table 3). The stoves were usually placed in the living room where most family members spent their time; very few households had separate stoves in the kitchen. The majority of students and adults were aware in a broad sense of the health effects of arsenic, especially in Xingren and Anlong counties (Table 4). About half the villages in these two counties surveyed in 2004 were part of an UNICEF-sponsored health education campaign that was conducted in 2003. Therefore, the high level of awareness was not entirely surprising, although our survey population was twice as large as that of the 2003 UNICEF education campaign. In Xingyi, where no recent health education campaign occurred, only one-third of the students and adults were aware of the health effects of As exposure (Table 4). Our questionnaire also revealed that many residents lacked knowledge of As exposure routes. Many residents were mistakenly attributing arsenicosis to drinking water or poor Fengshui. Many did not understand the relationship between unventilated indoor coal-burning stoves and skin lesions, cancers, and liver disease.

### Effect of mitigation, including health education

The health education campaign in 2005 resulted in an increased awareness in students and heads of households to approximately 90 and 85%, respectively. More important, the target population had a better understanding of As exposure from burning coal. Most of these individuals understood that when they do not use high-As coal and use ventilated stoves with chimneys that remove smoke from the room, the prevalence of arsenicosis can be reduced.

Two measures were implemented to reduce and eventually eliminate As exposure. Coal mines producing high-As coal were shut down. Executive orders prohibiting mining of local coal pits were posted to remind the villagers to observe the regulations. By the end of 2005, permanent signs warning of the risks of mining coal were placed at all coal pits known produce high-As coal. More important, 10,000 stoves with chimneys were installed between May and November of 2005. This changed the tradition of using unventilated stove to burn coal in the arsenicosis-endemic region. As part of the health education campaign, our team members visited 292 randomly selected households in three counties to observe behavior changes. The use of unventilated stoves in the three endemic counties decreased to approximately 10% (Table 5). Drying corn and chili peppers over unventilated stoves decreased to 9 and 7%, respectively (Table 5). Now, residents no longer dry corn over stoves in the endemic regions of Anlong and Xingyi counties; instead they dry them outdoors in the sun. In Anlong and Xingyi, most villagers replaced corn with other staple foods such as rice and wheat for reasons we do not completely understand.

Consistent with the behavior change before and after mitigation and the health education campaign of 2005, urinary As concentrations in villagers residing in the As-endemic area decreased (Table 6). In villagers without any symptoms of arsenicosis (i.e., control group), urinary As concentrations decreased by a factor of 3, from 0.045 ± 0.046 mg/L (*n* = 40) to 0.017 ± 0.007 mg/L (*n* = 10). This decrease is not statistically significant (*t* = 1.90, *p* > 0.05). In villagers with any symptom of arsenicosis (i.e., patient group), urinary As concentrations decreased by a factor of 4, from 0.198 ± 0.300 mg/L (*n* = 144) to 0.049 ± 0.009 mg/L (*n* = 50). This large decrease of urinary As in arsenicosis patients is statistically significant (*t* = 3.51, *p* < 0.01).

## Discussion

Our experience demonstrates that grass root health education specific to addressing local needs and knowledge gaps is the key to the success of our mitigation effort. Previous mitigation efforts in the region took place in the endemic regions, often in various forms but was met with limited success. There is some evidence of success in mitigation in Anlong and Xingyi. Endemic arsenicosis in these two counties was discovered in the 1970s. In the past decade, the number of households using high-As coal decreased significantly. The number of the arsenicosis cases decreased significantly from 640 in 1994 to 561 in 2004, excluding those individuals who died from arsenicosis (An and Li 2005; Li and An 2005). In Anlong county, two villages recently identified as having arsenicosis had only a few families that used high-As coal (Table 1). In Xingren, where arsenicosis is most severe (Table 1), previous mitigation has not been effective. This can be attributed partly to the lack of in-depth understanding by the villagers that using domestic coal can cause arsenicosis. Clearly, this was evident in our baseline survey data in 2004, which showed that high-As coal was still mined and used despite the apparent increased awareness of the health effects of As. Consequently, more and more villages continued to mine coal until very recently, and the number of arsenicosis cases increased to 2,811 in a population of 39,315 in 2004 from 2,205 in a population of 30,535 in 1994 (Table 1).

The arsenicosis endemic in Guizhou province is essentially a lifestyle disease related to poverty. Abundant coal from mines in the region is a source of free fuel for villagers who cannot afford commercial coal or other fuels for heating and cooking. Our study shows that villagers were willing to change their lifestyles after the link between exposure to high-As coal and arsenicosis was established through health education, especially when the entire community was mobilized. But it is also important to point out that a concerted mitigation effort by all stakeholders, including many government agencies, and proper financing are also important to the success of providing the basis for lifestyle changes for villagers. In other words, the mitigation plan must be comprehensive and address source elimination through the closing of coal mines, lifestyle changes that enable the use of ventilated stoves and other means of drying corn and chili peppers, and finally, through health education that reinforces the positive benefits of the new lifestyles. This understanding occurred during a pilot study conducted in 2000 (An et al. 2001b). We extended and applied the tested approach of intervention and were able to rapidly raise awareness and the level of the related knowledge among the residents of the arsenicosis-endemic regions through a mitigation effort that lasted only about 6 months (An et al. 2001a; Li et al. 2005a). Today, mining of high-As coal has all but ceased, and nearly all the affected households are using the improved stoves with ventilation. Some residents set up biomass pool and biogas stoves, replacing coal with renewable biofuel.

We cannot be content with the initial success of reduction of As exposure indicated by the behavior changes and reduction of urinary As concentrations. Continued health education and monitoring are required to ensure that the lifestyle changes are sustainable. In addition, for nearly 3,000 individuals with arsenicosis in the endemic area in Guizhou province, many have developed advanced skin lesions such as hyperkeratosis and have lost the ability to work and earn income. Although the source of As exposure is now largely eliminated, cumulative exposure from past decades indicates that new cases of arsenicosis will continue to emerge in the decades to come. How to provide better health care and treatment to the people of Guizhou province in order to reduce the occurrence of arsenicosis and the mortality rate from malignant tumors remains challenging for years to come (Li et al. 2002).

## Figures and Tables

**Table 1:** Occurrence of arsenicosis in three counties in Guizhou, China, in 1994 and 2004.

	No. of endemic towns		No. of endemic villages		Population (*n*) in endemic villages		No. of arsenicosis patients	
Endemic county	1994	2004	1994	2004	1994	2004	1994	2004
Xingyi	2	2	5	5	2,881	4,153	214	177
Xingren	1	3	9	13	14,685	20,747	1,565	2,250
Anlong	2	3	11	13	12,969	14,415	426	384
Total	5	8	25	31	30,535	39,315	2,205	2,811

**Table 2:** Arsenic concentrations in coal, food, and indoor air in Guizhou, China.

	Arsenic in:			
Endemic county	Coal (mg/kg)	Unventilated stove-dried corn (mg/kg)	Unventilated stove-dried chili (mg/kg)	Indoor air (mg/m^3^)
Xingren^a^	418 ± 530 (13)	4 ± 3 (70)	512 ± 300 (71)	0.3 ± 0.2 (37)
Xingyi	265 ± 352 (14)	7 ± 12 (20)	694 ± 545 (20)	—
Anlong	624 ± 852 (24)	7 ± 12 (31)	688 ± 586 (31)	0.1 ± 0.1 (21)

Values are means ± SD; *n* = number of samples.

a As concentrations in coal, corn, chili, and indoor air in the nonendemic area (control) of Xingren are 11.2 ± 2.9 (*n* = 6) mg/kg, 0.36 ± 0.11 (*n* = 20) mg/kg, 0.39 ± 0.46 (*n* = 20) mg/kg, and 0.045 ± 0.041 (*n* = 6) mg/m^3^, respectively (Li et al. 2000).

**Table 3:** Behavior related to arsenic exposure before mitigation in 2005 in Guizhou, China.

County	No. of households surveyed	Use of unventilated stoves without chimneys (%)	Use of unventilated stoves to dry corn (%)	Use of unventilated stoves to dry chili peppers (%)	Cooking corn without washing (%)	Cooking chili peppers without washing (%)
Xingyi	5,000	96	88	82	95	83
Xingren	2,368	98	88	89	96	97
Anlong	4,186	95	80	100	100	100
Total	11,554	96	87	89	97	96

**Table 4:** Awareness of health effects of arsenic exposure before and after health education in 2005 in Guizhou, China.

	2004 baseline survey				2005 survey			
County	Population (*n*)	Student awareness (%)	Population (*n*)	Adult awareness (%)	Population (*n*)	Student awareness (%)	Population (*n*)	Adult awareness (%)
Xingyi	200	36	80	34	200	87^a^	80	88^b^
Xingren	264	89	92	56	264	98^c^	92	85^d^
Anlong	349	80	120	70	349	89^e^	120	84^f^
Total	813	68	292	53	813	91	292	85

a χ^2^ = 107.71, *p* < 0.001.

b χ^2^ = 48.69, *p* < 0.05.

c χ^2^ = 17.63, *p* < 0.0001.

d χ^2^ = 16.38, *p* < 0.001.

e χ^2^ = 9.08, *p* < 0.01.

f χ^2^ = 6.04, *p* < 0.05.

**Table 5:** Changes in behavior related to arsenic exposure after mitigation in 2005 in Guizhou, China.

County	No. of visited households	Use of unventilated stove without chimney (%)	Use of unventilated stove to dry corn (%)	Use of unventilated stove to dry chili pepper (%)	Cooking corn without washing (%)^a^	Cooking chili peppers without washing (%)
Xingyi	92	1	9	4	—	23
Xingren	80	18	9	5	43	33
Anlong	120	9	8	9	—	44
Total	292	10	9	7	—	32

a The villagers in the Xingyi and Anlong endemic regions no longer include corn in their diet.

**Table 6:** Urinary arsenic concentrations (mg/L) in exposed population before and after mitigation in 2005 in Guizhou, China.

	2004 baseline survey			2005 survey		
Subject	No. of cases	Mean ± SD	Range	No. of cases	Mean ± SD	Range
Control	40	0.045 ± 0.046	0–0.16	10	0.017^c^ ± 0.007	0–0.05
Patient	144	0.198^a^ ± 0.300	0.001–1.73	50	0.049^b^ ± 0.009	0.001–0.326

a *t* = 3.21, *p* < 0.01 (compared with controls).

b *t* = 3.51, *p* < 0.001 (compared with the group of arsenicosis patients before intervention).

c *t* = 1.90, *p* > 0.05 (compared with the controls surveyed in 2004).
